# Biomarkers for Dementia, Fatigue, and Depression in Parkinson's Disease

**DOI:** 10.3389/fneur.2019.00195

**Published:** 2019-03-08

**Authors:** Tino Prell, Otto W. Witte, Julian Grosskreutz

**Affiliations:** ^1^Department of Neurology, Jena University Hospital, Jena, Germany; ^2^Center for Healthy Ageing, Jena University Hospital, Jena, Germany

**Keywords:** Parkinson's disease, biomarker, non-motor syndromes, depression, fatigue, dementia

## Abstract

Parkinson's disease is a common multisystem neurodegenerative disorder characterized by typical motor and non-motor symptoms. There is an urgent need for biomarkers for assessment of disease severity, complications and prognosis. In addition, biomarkers reporting the underlying pathophysiology assist in understanding the disease and developing neuroprotective therapies. Ultimately, biomarkers could be used to develop a more efficient personalized approach for clinical trials and treatment strategies. With the goal to improve quality of life in Parkinson's disease it is essential to understand and objectively monitor non-motor symptoms. This narrative review provides an overview of recent developments of biomarkers (biofluid samples and imaging) for three common neuropsychological syndromes in Parkinson's disease: dementia, fatigue, and depression.

## Introduction

Parkinson's disease (PD) is now considered as progressive and multisystem α-synucleinopathy. Therefore, PD is characterized not only by motor symptoms, but also a broad range of non-motor symptoms (NMS) (1). NMS can aggravate disease burden and significantly contribute to worsening of quality of life (2). Biomarkers which are associated with worse motor performance as well as development of NMS are of special importance in PD. A biomarker is “a characteristic that is objectively measured and evaluated as an indicator of normal biological processes, pathogenic processes, or pharmacologic responses to a therapeutic intervention” (3). The ideal PD biomarkers should have a reasonable effect size, are reproducible across different cohorts and are ideally verified in neuropathological proven PD cases. Biomarkers in PD can include (i) biomarker for prodromal stage to identify PD before motor symptoms occur, (ii) biomarkers of susceptibility to identify persons who are at risk for PD, (iii) biomarkers for motor and non-motor burden to assess disease severity and monitor the efficacy of therapies. The last one can help to identify patients who are at risk to develop complications and may lead to individual optimization and prevention in health care. This review provides an update on recent advances in the development of biomarkers (biofluid samples and neuroimaging) for three common neuropsychological syndromes: dementia, fatigue and depression.

## Cognitive Impairment

Cognitive deficits are common in PD and can present as mild dysfunction in the prodromal and early stages, or as dementia (PDD) in advanced stages (4). Approximately 20% of patients with *de novo* PD have mild cognitive impairment (MCI) (5). The concept of PD-MCI was introduced 2012 (MDS Task Force) and characterizes a cognitive decline that is assessed during neuropsychological testing but does not impair activities of daily living (6). MCI is considered an intermediate state of cognitive dysfunction in PD that may progress to PDD. Up to 75% of patients will develop dementia over the longterm disease course (7). However, the rate to PDD, the cognitive profile and severity of cognitive dysfunction show high interindividual variation. Given its high medical and social impact and its health-related costs, the identification of biomarkers for PDD is of high priority (8). Biomarkers reflecting cognitive decline can facilitate early diagnosis and may indicate response to therapeutic interventions.

Clinical factors, such as higher age, male sex, low level of education, longer disease duration, higher Hoehn & Yahr stage, axial impairment, excessive daytime sleepiness, cardiovascular autonomic dysfunction, REM sleep behavior disorder, hallucinations and PD-MCI were found to strongly predict the development of PDD (9–13). Moreover, impairment of memory and language (posterior-cortical dysfunction) seems to be linked to a higher risk of PDD (14, 15).

Given the neuropathology of PDD several studies aimed to identify biomarkers which reflect proteinopathy, neuronal loss, abnormal neurotransmitters, and structural and functional brain changes. Lewy bodies and amyloid plaques in the neocortex and limbic system are typical neuropathological features of Alzheimer's disease and PDD (16, 17). Hence, the majority of studies investigated amyloid-ß 1–42 (Aß), tau protein, and α-synuclein in the cerebrospinal fluid (CSF) of PD patients (Table 1). In many studies the level of Aß was reduced in PDD. Low CSF levels of Aß were found to be related to deterioration in attention, executive function, semantic fluency and memory (21, 38, 40, 45). One-half of PDD patients had the CSF biomarker signature of Alzheimer's disease (46) suggestive of an overlap with Alzheimer's disease pathology (47). Low baseline CSF Aβ was associated with more rapid cognitive decline later in disease. By contrast, the levels of total (t-tau) and phosphorylated tau (p-tau) were found to be increased or unchanged in PDD (Table 1). For clinicians it is highly relevant to know which biomarkers accurately predict the progression from MCI to PDD. Therefore, based on the data from cross-sectional and longitudinal studies one can assume that reduced Aß predicts cognitive decline in PD (40, 42, 48).

**Table 1:** Cerebrospinal-fluid (CSF) biomarkers of cognitive impairment and dementia in Parkinson's disease.

**Study**	**CSF biomarker**						**Participants**	**Methods**	**Result**
	**Aß1-42**	**t- tau**	**p-tau**	**t-α-syn**	**o-α-syn**	**other**			
Alves et al. (18)^*^	+	+	+				PDND 104	MDS Task Force	Low Aβ predicted early dementia
Bäckström et al. (19)^*^	+	+	+	+		+	PDND 104 C 30 PSP 13 MSA 11	NFL H-FABP	Low Aβ, NFL and H-FABP predicted PDD
Brockmann et al. (20)	+					+	PDND 353 PDD 103	Genetic variants known to be involved in Aβ clearance	Risk variants in *APOE* and cystatin C genes were associated with lower Aβ
Compta et al. (21)	+	+					PDND 20 PDD 20 C 30	MMSE DSM-IV-R MDS Task Force	PDD: ↑ t-tau PDND: ↓ Aβ positively correlated with phonemic fluency
Compta et al. (22)	+	+	+				PDND 19 PDD 29 C 9	MMSE MDS Task Force	PDD: ↓ Aβ ↑ t-tau and p-tau in a subgroup
Compta et al. (23)^*^	+	+	+				PDND 27	MMSE MDS Task Force	Low Aβ predicted PDD
Compta et al. (24)	+	+		+	+		PDND 21 PDD 20 C 13	MMSE/PDD by MDS Task Force	PDD: ↓ Aβ, ↑ t-tau, ↑ o-α-syn
Ffytche et al. (25)	+						PD 423 3-4 years of follow-up	Compare baseline structural imaging and CSF data in patients who go on to develop illusions or hallucinations in newly diagnosed PD	Patients with early onset PD psychosis: Aβ ↓
Gmitterová et al. (26)	+	+	+			+	PDND 22 PDD 31 DLB 51 C 32	Discriminatory potential of tau, p-tau, Aβ, NSE and S100B across the spectrum of LBD	PDD Aβ ↓, tau ↑ Rapid disease course not associated with decrease of Aβ
Halbgebauer et al. (27)						+	PDND 22 PDD 29 C 36	Modified serpinA1	PDD: acidic serpinA1 isoform ↑
Hall et al. (28)	+	+	+	+			PDND 90 PDD 33 C 107	MMSE MDS Task Force	PDD: ↑ p-tau, Aβ or t-α-syn no differences
Hall et al. (29)^*^	+	+	+	+		+	PDND 42 C 69		Low Aβ predicted memory decline, high α-syn predicted reduced cognitive speed
Hansson et al. (30)				+	+		PDND 30 C 98	MMSE MDS Task Force	PDD: ↑ o-α-syn
Janssens et al. (31)	+	+	+			+	probable AD 52 FTD 59 DLB 39 PDD 14 C 88 young C 32	3-methoxy-4-hydroxyphenylglycol (MHPG)	Aβ young C > C > FTD > PDD, DLB > AD tau AD > FTD > PDD, DLB > C > young C p-tau AD > FTD = PDD,DLB = C> young C MHPG PDD, DLB > AD > C
Lindqvist et al. (32)						+	PDND 71 PDD 16 C 33	MMSE	PDD: C-reactive protein ↑ IL6 ↑ TNF-Alpha → Eotaxin → MCP-1 → MIP-1beta → IP-10 →
Maetzler et al. (33)	+						PDND 14 PDD 12	MMSE	PDD: Aβ ↓
Maetzler et al. (34)	+	+					PDND 21 PDD 10 C 39	MMSE	No difference
Maetzler et al. (35)	+	+					PDND 77 PDD 26 C 72	MMSE MDS Task Force	No difference
Modreanu et al. (36)	+	+	+				PD 37 PDD 21 PDD at 18-months 35	Spatial disorientation, memory complaints over disease course	PDD: Aβ ↓ tau and p-tau no difference ‘PDD -converters' had significantly lower Aβ at baseline
Parnetti et al. (37)		+	+				PDND 67 PDD 48 C 41	MMSE	No difference
Parnetti et al. (38)^*^	+	+	+	+	+		PDND 44 Disease C 25	MMSE MoCa	Low Aβ predicted more rapid decline
Schrag et al. (39)^*^	+	+					PDND 390 C 178	MoCa over 2 years	Low Aβ/t-tau ratio predicts cognitive decline
Siderowf et al. (40)^*^	+	+	+				PDND: 45	Dementia rating scale	Low Aβ predicted rapid decline in Dementia rating scale
Stewart et al. (41)^*^	+	+	+	+			PDND 350	Verbal memory, cognitive processing speed, and visuospatial working memory	Lower α-synuclein predicted better preservation of cognitive function
Terrelonge et al. (42)^*^	+	+	+	+			PDND 341	Memory, visuospatial, working memory–executive function, and attention processing speed	Low Aβ predicted cognitive impairment
Vranová et al. (43)	+	+					PDND 27 PDD 14 C 14	MMSE MDS Task Force	PDD: ↑ t-tau/ Aβ index Aβ or t-tau no differences
Wennström et al. (44)				+			PDND 38 PDD 22 C 52	MMSE MDS Task Force	No difference

PD, Patients with Parkinson's disease; PD-MCI, Parkinson's disease patients with mild cognitive impairment; PDD, Parkinson's disease patients with dementia; PDND, non-demented PD; MSA, multiple system atrophy; PSP, progressive supranuclear palsy; AD, Patients with Alzheimer's disease; DLB, Dementia with Lewy body; C, Controls; MoCA, Montreal Cognitive Assessment; MMSE, Mini Mental Status Examination; Aβ, Aβ_1−42_ amyloid; NFL, neurofilament light chain protein; H-FABP, heart fatty acid-binding protein;

* ***longitudinal studies***.

Several studies assessed the CSF levels of α-synuclein in PD. Meta-analyses demonstrated that total α-synuclein levels are lower in PD compared to controls (49, 50). However, in terms of α-synuclein and cognitive decline there are conflicting results with both low and high levels in the presence of cognitive impairment (29, 41, 48). In the DATATOP study with up to 8 years of follow-up, lower α-synuclein levels predicted better preservation of cognitive function (verbal learning and memory, visuospatial working memory) in early disease. Thus, α-synuclein may reflect changes in multiple cognitive domains and may predict cognitive decline in PD (29, 41, 48). On the other hand most studies of non-demented PD failed to find any association between α-synuclein levels and cognition (51, 52). It seems that CSF α-synuclein levels may increase with disease stage. This could explain why cognitive deficits in connection with high levels of α- synuclein were found in more advanced disease stages (53). Isoforms of α-synuclein (e.g., phosphorylated, ubiquitinated, oligomeric) are potentially more sensitive to cognitive decline than the total α-synuclein level (24, 30). Another study examining plasma levels of α-synuclein found higher levels in PDD and a correlation with mini mental state examination scores (54). This finding, however, requires further investigations.

In another longitudinal study, high neurofilament light chain protein, low Aβ and high heart fatty acid–binding protein at baseline were related to future PDD with a relatively high diagnostic accuracy (19). Also several serum proteins, such as C-reactive protein, interleukins, interferon-γ, tumor necrosis factor α, uric acid, and cystatin C were found to be associated with cognition in PD (55). In particular, low uric acid concentrations, low levels of epidermal growth factor (EGF) and insulin-like growth factor (ILGF) seems to have predictive value for deterioration of cognitive function in PD (56–61). In combination with clinical markers, a study of 390 patients from the Progression Markers Initiative study with newly diagnosed PD, the occurrence of cognitive impairment at 2 years follow-up could be predicted with good accuracy using a model combining information on age, non-motor assessments, DAT imaging, and CSF biomarkers. Here, the Montreal Cognitive Assessment (MoCA) scores and low CSF Aβ to t-tau ratio and DAT imaging results were the best predictors of cognitive impairment (39). Using data from the Parkinson's Progression Markers Initiative, Fereshtehnejad et al., identified distinct subgroups via a cluster analysis of a comprehensive dataset consisting of clinical characteristics, neuroimaging, biospecimen and genetic information. Here, the CSF biomarkers differed between these PD subtypes. Patients with diffuse malignant disease course and fast cognitive decline, showed an Alzheimer's disease-like CSF profile (low Aβ, low Aβ/t-tau ratio) (62).

Applying computerized neuroimaging analyses several MRI studies have found gray matter atrophy and disruptions of white matter integrity in PDD, although findings in non-demented PD and PD-MCI remain inconsistent (63) (Tables 2, 3). A longitudinal study using voxel-based morphometry (VBM) found neocortical volume reduction (temporo-occipital region, hippocampal and parahippocampal) as the most relevant finding in patients who develop PDD (97). Another study has identified a validated Alzheimer's disease pattern of brain atrophy as an independent predictor of cognitive impairment in PD (64). More specifically cortical thinning in the right precentral, frontal, and in the anterior cingulate cortex as well as gray matter atrophy (prefrontal, insula, caudate nucleus, hippocampal) predicted cognitive decline in PD (23, 66, 70, 76, 98). Cognitive impairment was also found to be associated with lower gray matter volume and increased mean diffusivity in the nucleus basalis of Meynert, compared to non-demented patients. Moreover, these changes were predictive for developing cognitive impairment in cognitively intact patients with PD, independent of other clinical and non-clinical markers of the disease (99). The nucleus basalis of Meynert and the pedunculopontine nucleus in the brainstem are important cholinergic projections in and post-mortem studies have shown that neuronal loss in in the nucleus basalis is an early phenomenon in PD (100, 101). Combining many modalities, Compta et al. (23) performed a longitudinal study in non-demented PD patients including CSF, neuropsychological and MRI studies at baseline and 18 months follow up. Here, a combination of lower CSF Aβ, reduced verbal learning, semantic fluency and visuoperceptual scores, as well as cortical thinning in superior-frontal/anterior cingulate and precentral regions were found to be predictive for PDD.

**Table 2:** Cortical and subcortical structural changes related to cognitive impairment and dementia in Parkinson's disease.

**Study**	**Participants**	**Methods**	**Result**
Weintraub et al. (64)	PDND 60	VBM^*^	In PD-MCI hippocampal and temporal gray matter atrophy.
Melzer et al. (65)	PDND 57 PD-MCI 23 PDD 16 C 34	VBM	In PD-MCI gray matter atrophy in temporal, parietal, frontal cortex, amygdala, right putamen, and hippocampus. In PDD additional atrophy in medial temporal lobe, lingual gyrus, posterior cingulate gyrus, and bilateral caudate.
Lee et al. (66)	PD-MCI 51 C 25	VBM^*^	PD-MCI to PDD converters had lower GM density in the left prefrontal areas, left insular cortex and bilateral caudate nucleus compared with that in PD-MCI non-converters.
Borroni et al. (67)	PDND11 PDD 10 LBD 13 C 10	VBM	In PDD bilateral frontal and subcortical (caudate nucleus) gray matter atrophy.
Duncan et al. (68)	PDND 125 C 50	VBM DTI	Frontal and parietal gray matter volume reductions were associated with reduced executive function. Increased mean diffusivity was associated with performance on the semantic fluency and Tower of London tasks in frontal and parietal white matter tracts.
Hattori et al. (69)	PDND 32 PD-MCI 28 PDD 25 DLB 29 C 40	VBM TBSS	In PDD more atrophy in the cerebellum, thalami, insula, parietal cortex and occipital cortex.
Kandiah et al. (70)	PDND 97	Hippocampal volume White matter hyperintensities^*^	Hippocampal volume predicts PD-MCI and PDD.
Rektorova et al. (71)	PDND 75 PD-MCI 29 PDD 22	Spatial Independent Component Analysis	In PDD gray matter volume reductions in the hippocampus and temporal lobes, fronto-parietal regions and increases in the midbrain/cerebellum correlated with visuospatial deficits and letter verbal fluency, respectively.
Biundo et al. (72)	PDND 15 PD-MCI 14 HC 21	Cortical thickness	In PD-MCI cortical thinning in right supramarginal, dorsolateral prefrontal cortex, hippocampus, orbito-frontal, fusiform, superior parietal, and cuneus.
Pereira et al. (73)	PDND 90 PD-MCI 33 H 56	Cortical thickness	In PD-MCI cortical thinning in left precuneus, inferior temporal precentral, superior parietal, and lingual regions.
Hanganu et al. (74)	PDND 15 PD-MCI 17 H 18	Cortical thickness ^*^	In PD-MCI thinning in temporal and medial occipital lobe, nucleus accumbens and amygdala correlate with cognitive decline.
Ibarretxe-Bilbao et al. (75)	PDND 16 C 15	Cortical thickness^*^	In PD cortical thinning in bilateral fronto-temporal regions and reduced amygdala volume.
Mak et al. (76)	PDND 66 PD-MCI 39 H 37	Cortical thickness^*^	PD-MCI converters showed bilateral temporal cortex thinning at baseline.
Hwang et al. (77)	PDND 12 PDD 11 C 14	Cortical pattern matching Cortical thickness	PDD showed thinning bilateral sensorimotor, lateral parietal, right posterior cingulate, parieto-occipital, inferior temporal and lateral frontal relative to C and PDND.
Zarei et al. (78)	Early PD 24 moderate PD 18 PDD 15 C 39	Cortical thickness	MMSE correlated positively with cortical thickness in the anterior temporal, dorsolateral prefrontal, posterior cingulate, temporal fusiform and occipitotemporal cortex.
Pagonabarraga et al. (79)	PDND 26 PD-MCI 26 PDD 20 C 18	Cortical thickness	From PDND to PDD a linear and progressive cortical thinning was observed in areas functionally specialized in declarative memory (entorhinal cortex, anterior temporal pole), semantic knowledge (parahippocampus, fusiform gyrus), and visuoperceptive integration (banks of the superior temporal sulcus, lingual gyrus, cuneus and precuneus).
Carlesimo et al. (80)	PDND 25 C 25	DTI	Increased mean diffusivity in the PD hippocampi; high hippocampal mean diffusivity values obtained low memory scores.
Chen et al. (81)	PDND 19 PDD 11 C 21	DTI	In PDD lower fractional anisotropy in the left hippocampus, higher mean diffusivity in widespread white matter regions. In PD positive correlation between MoCA score and fractional anisotropy of left inferior longitudinal and hippocampus, and bilateral superior longitudinal fasciculus.

PD, Patients with Parkinson's disease; PD-MCI, Parkinson's disease patients with mild cognitive impairment; PDD, Parkinson's disease patients with dementia; PDND, non-demented PD; DLB, Dementia with Lewy body; C, Controls; MoCA, Montreal Cognitive Assessment; MMSE, Mini Mental Status Examination;

* ***longitudinal studies***.

**Table 3:** Changes of function and connectivity related to cognitive impairment and dementia in Parkinson's disease.

**Study**	**Participants**	**Methods**	**Result**
Gorges et al. (82)	PDND 14 PDD 17 C 22	Resting-state fMRI	In PDND hyperconnectivity (network expansions) in cortical, limbic, and basal ganglia-thalamic areas. In PDD decreased intrinsic functional connectivity compared with controls (predominantly between major nodes of the default mode network).
Baggio et al. (83)	PDND 32 PD-MCI 23 C 36	Resting-state fMRI	In PD-MCI reduced connectivity between dorsal attention network and right fronto-insular regions (worse performance in executive functions) and increased connectivity between default mode network and medial and lateral occipito-parietal regions (worse visuo-spatial performance).
Amboni et al. (84)	PDND 21 PD-MCI 21 C 20	Resting-state fMRI	In PD-MCI patients decreased functional connectivity in bilateral prefrontal cortex (fronto-parietal network).
Tessitore et al. (85)	PDNT 16 C 16	Resting-state fMRI	In PDND decreased default mode network connectivity correlated with cognitive parameters.
Rektorova et al. (86)	PDND 18 PDD 14 C 18	Resting-state fMRI	In PDD decreased connectivity in the right inferior frontal gyrus compared to PDND and C (using posterior cingulate cortex/precuneus as seed for analysis).
Borroni et al. (67)	PDND11 PDD 10 LBD 13 C 10	Resting-state fMRI	Reduced local coherence of frontal regions in PD and in PDD.
Olde et al. (87)	PDND 55 C 15	Resting-state fMRI	In PDND longitudinally decreases in functional connectivity most prominent for posterior brain regions correlated with disease progression and cognitive decline.
Seibert et al. (88)	C 19 PDND 19 PDD 18	Resting-state fMRI	In PDD corticostriatal functional correlations were decreased in bilateral prefrontal regions.
Lin et al. (89)	PDND 17 PDD 17 C 17	Arterial spin labeling (ASL) magnetic resonance imaging (ASL-MRI)	In PDND and PDD progressive widespread cortical hypoperfusion.
Le Heron et al. (90)	PDD 20 AD 17 C 37	Arterial spin labeling (ASL) magnetic resonance imaging (ASL-MRI)	In AD and PDD posterior hypoperfusion (including posterior cingulate gyrus, precuneus, occipital regions). Perfusion in medial temporal lobes (AD < PDD) and right frontal cortex (PDD < AD) differed between PDD and AD.
Vander Borght et al. (91)	PDD 9 AD 9 C 9	[18F]fluorodeoxyglucose-PET	In PDD and AD hypometabolism with similar regional accentuation (lateral parietal, lateral temporal and lateral frontal association cortices and posterior cingulate cortex). In contrast to AD PDD showed greater metabolic reduction in the visual cortex and relatively preserved metabolism in the medial temporal cortex.
Gonzalez-Redondo et al. (92)	PDND 14 PD-MCI 17 PDD 15 C 19	[18F]fluorodeoxyglucose-PET	In PD-MCI the hypometabolism exceeded atrophy in the angular gyrus, occipital, orbital and anterior frontal lobes. In PDD these areas were atrophic and surrounded by extensive hypometabolism.
Shinotoh et al. (93)	PDND 14 PDD 2 PSP 12 C 13	Acetylcholinesterase activity using N-methyl-4-[11C]piperidyl acetate PET	In PDD higher reduction of choline acetyltransferase and acetylcholinesterase than in PDND.
Bohnen et al. (94)	PDND 11 PDD 14 AD 12 C 10	Acetylcholinesterase activity using [^11^C]Methylpiperidin-4-ylpropionate PET	Mean cortical acetylcholinesterase activity was lowest in PDD.
Hiraoka et al. (95)	PDD 12 C 13	[5-(11)C-methoxy]donepezil-PET	In PDD density of acetylcholinesterase in the cerebral cortices correlated with improvements in visuoperceptual function after 3 months of donepezil therapy.
Kotagal et al. (96)	PDND 11 PDD 6 DLB 6 C 14	Acetylcholinesterase activity using [^11^C]Methylpiperidin-4-ylpropionate PET	Thalamic cholinergic denervation is present in PD, PDD, and DLB but not in AD.

*PD, Patients with Parkinson's disease; PD-MCI, Parkinson's disease patients with mild cognitive impairment; PDD, Parkinson's disease patients with dementia; PDND, non-demented PD; DLB, Dementia with Lewy body; AD, Patients with Alzheimer's disease; C, Controls; MoCA, Montreal Cognitive Assessment; MMSE, Mini Mental Status Examination; PET, positron emission tomography*.

For the assessment of white matter pathology using DTI and imaging of metabolites (Proton magnetic resonance spectroscopy) there is currently not enough longitudinal data available and the value of these techniques to predict cognitive decline has to be further explored. The existing studies indicate that microstructural changes, such as increased mean diffusivity or reduced fractional anisotropy in the hippocampus, the frontal and parietal white matter tracts are associated with cognitive decline in PD (68, 80, 81, 102–104). In particular, an increased mean diffusivity may be predictive for cognitive decline before fractional anisotropy decreases. However, these findings need further validation in longitudinal studies.

## Fatigue

Fatigue is a common symptom that includes both mental and physical aspects. Up to 70% of individuals with PD experience fatigue every day (105). Fatigue dramatically impairs quality of life (106). It is a complex syndrome emerging from dysfunction in the nervous, endocrine and immune system (107). From a clinical point of view fatigue is frequently associated with other non-motor syndromes, like sleepiness, apathy, depression and autonomic dysfunction (105, 108). However, fatigue can also occur as an isolated syndrome; it is therefore important to understand that fatigue and sleepiness or depression is not the same condition (109, 110). Central fatigue is commonly measured through questionnaires, such as the Fatigue Severity Scale (111) which is recommended by the Movement Disorder Society (MDS) task force (112). Central fatigue can be described as a feeling of constant exhaustion and can occur in various chronic disorders. Peripheral fatigue is characterized by failure to sustain the force of muscle contraction and is more readily accessible to quantification (106, 113).

A key mechanism underlying fatigue is the activation of the inflammatory cytokine network (107, 114). Therefore, inflammatory markers serve as potential biomarkers of fatigue. In particular, higher serum levels of IL-6, IL1-Ra, sIL-2R, and VCAM-1 were associated with higher fatigue levels in patients with newly diagnosed, drug-naïve PD (115, 116). This neuroinflammatory processes may promote glutamate dysregulation and further influence neuronal activity and neuroplasticity, and impact neuronal circuits mediating distress and motivation in PD (117–119). Interestingly, higher serum uric acid levels were significantly associated with less fatigue (120).

In addition, dysfunction of the endocrine system, such as hypothalamic-pituitary-adrenal system which is connected to basal ganglia, amygdala, thalamus and frontal cortex, seems to contribute to the pathophysiology of fatigue (113). Although there are no neuropathological studies of PD-fatigue supporting this model so far, several neuroimaging studies showed that multiple brain areas are involved in fatigue in PD. These include frontal, temporal and parietal regions indicative of emotion, motivation and cognitive functions (121–126). In SPECT imaging with technetium-99 hexamethyl-propylene-amine-oxime PD-fatigue was associated with reduced perfusion in the frontal lobe (125). Others used PET with dopaminergic and serotonergic markers in fatigued vs. non-fatigued PD patients. Less serotonergic marker binding was found in striatal and limbic regions (thalamus, anterior cingulate, amygdala, insula) in PD-fatigue. The striatal ^18^F-dopa uptake was similar in fatigued and non-fatigued groups, but voxel-based analysis localized the reduced dopamine uptake to the caudate and insula in PD-fatigue (127). In addition the serotonin transporter (SERT) availability was significantly reduced in the striatum and thalamus of fatigued PD patients, suggesting that increasing the brain level of serotonin may improve PD-fatigue (127). The reduced serotonergic transmission suggests that a disturbed neurotransmitter balance within the basal ganglia and associated regions changes the integration of emotional and motor information in limbic regions, thus resulting in fatigue symptoms (128). With regard to striatal dopamine transporter uptake, results are conflicting. Two studies found no difference between fatigued and non-fatigued PD (127, 129). In the study by Chou et al., striatal dopamine transporter uptake was a significant predictor of fatigue in mild but not moderate-to-severe PD. They postulated that the lack of association between fatigue and nigrostriatal loss in advanced PD may reflect a denervation “floor” effect (130). Many of these studies have assessed advanced disease stages and patients on dopaminergic treatment. In contrast, Tessitore et al. studied fatigue in drug-naïve early PD using resting-state functional MRI (fMRI). Fatigue itself, and fatigue severity were associated with a decreased connectivity within the supplementary motor area and an increased connectivity within the default mode network (121). Importantly, these functional abnormalities occur independently from both dopamine-induced connectivity and structural changes. This study is in line with earlier neurophysiological studies suggesting that abnormal premotor and primary motor cortices connectivity correlate with fatigue (131, 132). Tessitore et al. hypothesized that the increased connectivity of the default mode network represents an initial cognitive compensatory response to the fatigue-related motor connectivity changes. In this sense fatigued PD-patients, when internally oriented, have to increase mental expenditure to maintain the same level of motor planning performance in order to switch more easily to externally oriented processing (121).

In summary, abnormalities in motivation of self-initiated tasks and motor function may play a significant role in the pathophysiology of fatigue (133). While non-dopaminergic basal ganglia pathways seem to be involved in PD-fatigue, the dopaminergic dysfunction may only play a role through extrastriatal projections.

## Depression

PD patients are twice as likely to develop depression compared to healthy individuals (134). Depressive symptoms affect 40–50% of PD patients and significantly impact quality of life in PD (2). In particular, patients with cognitive impairment, longer disease duration, motor fluctuations, female gender, and higher doses of levodopa are at risk to develop depression (9).

Like other NMS, depression seems to be linked to inflammatory signaling. Increased inflammatory responses have been described both in the brain and peripheral blood of PD patients (135). Depression correlated with a high serum level of IL-10 (136) and IL-6 (137). High levels of both sIL-2R and TNF-α in blood samples from PD patients were significantly associated with more severe depression and anxiety (119). As reflection of CNS involvement, high CRP levels in CSF of PD patients were associated with more severe symptoms of depression (32). However, these findings are not specific for PD. Chronic inflammation in physically ill patients is often associated with symptoms of depression and also occurs in normal aging (138–140). Moreover, PD in general is characterized by elevated levels of inflammatory cytokines, such as IL-6, tumor necrosis factor, IL-1β, IL-2, IL-10, C-reactive protein, and RANTES (141).

Depression in PD is associated with several structural and functional changes in the limbic system. In particular, changes in the amygdala, hippocampus and orbitofrontal cortex were frequently reported in PD depression (142–151). The involvement of the serotonergic system was demonstrated in post-mortem tissue and validated *in vivo* by several PET imaging studies (152–155). Compared to controls the serotonin transporter binding in non-depressed PD was lower in the striatal region, the orbitofrontal cortex, and the dorsolateral pre-frontal cortex which is an area known to be involved in major depression (155). Using dopaminergic and serotonergic presynaptic transporter radioligands a prominent role of serotonergic degeneration in limbic regions such as the anterior cingulate cortex was demonstrated (156, 157). Other PET studies observed a higher availability of the serotonin transporter in the raphe nuclei and limbic regions of depressed PD patients (152, 153). Likewise, decreased plasma levels of serotonin were found to be correlated with severity of depression (158). However, studies of the serotonin metabolite 5-hydroxyindoleacetic acid (5-HIAA) in CSF from depressed and non-depressed PD patients, have yielded contradictory results (159), and serotonergic dysfunction alone may only explain vulnerability to depression in PD. Yet, symptoms of depression are also linked to mesolimbic dopaminergic degeneration (160, 161) which is in line with the clinical observation of improvement of depression by dopaminergic treatment (162).

## Conclusion

From this overview emerges a comprehensive picture of recent fluid and imaging biomarkers which have been studied in a number of clearly defined and sizable cohorts of PD patients with PD. Especially longitudinal studies are necessary to make the biomarkers potentially useful for therapeutic or even clinical trial evaluation. A number of recent studies have provided ample evidence for specific predictive biomarkers across multiple domains combining clinical, biochemical, and neuroimaging information. Yet, at this stage a lack of standardized and comparable methods preclude clinical everyday use of these biomarkers beyond their value as diagnostic or prognostic tools in cohorts of patients. Thus, more research needs to be undertaken into finding reliable combinations of predictors of NMS in PD on an individual level, and standardization and harmonization of protocols in particular in CSF handling and neuroimaging has to be taken further.

## Author Contributions

TP and JG: conception, collection of data, interpretation of data, drafting the work; OW: revising the work critically for important intellectual content.

### Conflict of Interest Statement

The authors declare that the research was conducted in the absence of any commercial or financial relationships that could be construed as a potential conflict of interest.
